# Calcium-dependent depletion zones in the cortical microtubule array coincide with sites of, but do not regulate, wall ingrowth papillae deposition in epidermal transfer cells

**DOI:** 10.1093/jxb/erv317

**Published:** 2015-07-01

**Authors:** Hui-ming Zhang, Mark J. Talbot, David W. McCurdy, John W. Patrick, Christina E. Offler

**Affiliations:** ^1^School of Environmental and Life Sciences, University of Newcastle, Callaghan, NSW 2308, Australia; ^2^CSIRO Agriculture, Canberra, ACT 2601, Australia

**Keywords:** Cortical microtubule arrays, cytosolic calcium, localized cell wall deposition, seed, transfer cell, wall ingrowth papillae.

## Abstract

*Trans*-differentiation to a transfer-cell morphology is characterized by the localized deposition of wall ingrowth papillae that protrude into the cytosol. Whether the cortical microtubule array directs wall ingrowth papillae formation was investigated using a *Vicia faba* cotyledon culture system in which their adaxial epidermal cells were spontaneously induced to *trans*-differentiate to transfer cells. During deposition of wall ingrowth papillae, the aligned cortical microtubule arrays in precursor epidermal cells were reorganized into a randomized array characterized by circular depletion zones. Concurrence of the temporal appearance, spatial pattern, and size of depletion zones and wall ingrowth papillae was consistent with each papilla occupying a depletion zone. Surprisingly, microtubules appeared not to regulate construction of wall ingrowth papillae, as neither depolymerization nor stabilization of cortical microtubules changed their deposition pattern or morphology. Moreover, the size and spatial pattern of depletion zones was unaltered when the formation of wall ingrowth papillae was blocked by inhibiting cellulose biosynthesis. In contrast, the depletion zones were absent when the cytosolic calcium plumes, responsible for directing wall ingrowth papillae formation, were blocked or dissipated. Thus, we conclude that the depletion zones within the cortical microtubule array result from localized depolymerization of microtubules initiated by elevated cytosolic Ca^2+^ levels at loci where wall ingrowth papillae are deposited. The physiological significance of the depletion zones as a mechanism to accommodate the construction of wall ingrowth papillae without compromising maintenance of the plasma membrane–microtubule inter-relationship is discussed.

## Highlight

In developing transfer cells, inward-directed deposition of wall ingrowth papillae into the cytoplasm occurs independently of, but through, depletion zones in the cortical microtubule array created by cytosolic Ca^2+^ plumes.

## Introduction

As part of their differentiation, numerous plant cell types deposit specialized cell walls that relate to their final function. Included among these cell types are transfer cells that *trans*-differentiate from many cell types, conferring on them an enhanced capacity for membrane exchange of nutrients. This functional state is achieved through the deposition of extensive ingrowth wall labyrinths that act as scaffolds to support an amplified surface area of plasma membrane enriched in nutrient transporters (Offler *et al.*, 2003). Ingrowth walls with a reticulate morphology comprise a uniform wall, deposited inward of the original primary wall, and from which wall ingrowth papillae arise at loci (McCurdy *et al.*, 2008; Andriunas *et al.*, 2013). Depending on the species and cell type, the papillae then branch and fuse, resulting in a fenestrated layer of wall material and, by repeating this sequence of wall deposition, form a multi-layered wall labyrinth (Talbot *et al.*, 2001). Such a multi-layered ingrowth wall labyrinth, polarized to their outer periclinal wall, characterizes the abaxial epidermal transfer cells of *Vicia faba* cotyledons. In contrast, their adaxial epidermal cells do not form transfer cells *in vivo*. However, when cotyledons are placed in culture on a Murashige and Skoog (MS) medium (Murashige and Skoog, 1962) lacking growth substances (Offler *et al.*, 1997), their adaxial epidermal cells spontaneously and rapidly (within hours; Wardini *et al.*, 2007) *trans*-differentiate into transfer cells that are morphologically (Talbot *et al.*, 2001) and functionally (Farley *et al.*, 2000) equivalent to their abaxial counterparts. This study reports the use of this cotyledon culture system to investigate whether microtubule organization plays a role in directing the construction of wall ingrowth papillae during transfer-cell *trans*-differentiation.

Plant cell walls are composites of many polymers, which are assembled and organized in a myriad of different configurations (Carpita, 2012). Microtubules, together with actin filaments, have key roles in cell wall construction, but as yet these are not fully understood (McKenna *et al.*, 2014). Microtubules typically assemble at the cell cortex, forming patterns ranging from co-aligned (parallel) to randomly organized arrays (Shaw, 2013). Early models of microtubule/cellulose microfibril interactions suggested a direct relationship between the spatial organization of cortical microtubules (CMTs) and alignment of cellulose microfibrils (Green, 1962; Ledbetter and Porter, 1963). While more recent studies (Himmelspach *et al.*, 2003; Sugimoto *et al.*, 2003; Paradez *et al.*, 2006) have demonstrated that, in growing cells, cellulose microfibrils can maintain their parallel trajectories when CMTs are depolymerized, the alignment theory remains strongly supported by new evidence. For instance, an anchor protein, POM2/CSI1, binds cellulose synthases, CMTs, and, potentially, the plasma membrane, and has been shown to ensure tracking of cellulose synthase complexes along CMTs for ordered cellulose microfibril deposition (Bringmann *et al.*, 2012; Lei *et al.*, 2013).

During cell differentiation, CMT positioning controls the patterning of deposition of specialized and localized walls, for example secondary walls in xylem tracheary elements (Oda *et al.*, 2010) and thickened inner walls of guard cells (Kong *et al.*, 2010). In these examples, reorganization of CMTs through interaction with specific plasma membrane proteins located in microdomains provides the template for localized cell wall deposition. This evidence, combined with the dependence of wall ingrowth formation on depositing a cellulose scaffold (Talbot *et al.*, 2007*a*), suggests that changes in the interaction between cellulose synthases and CMTs may well direct construction of wall ingrowth papillae at right angles to the original primary wall of transfer cells. The spiral intertwined cellulose microfibrils of the cellulose scaffold (Talbot *et al.*, 2007*a*) are consistent with cellulose synthase complexes being constrained at the tips of wall ingrowth papillae (see also model in McCurdy *et al.*, 2008). In this case, the role for CMTs would differ fundamentally from that in tip growing systems of root hairs and pollen tubes. Here, CMTs are excluded from the growing tip (Mendrinna and Persson, 2015) at which cellulose microfibrils are deposited in a randomized pattern (Akkerman *et al.*, 2012). While the role of actin is not the focus of this study, it is likely to be responsible for trafficking secretory vesicles containing cellulose synthases to, but not constraining cellulose synthases at, the tips of developing wall ingrowth papillae, a role equivalent to that in tip growing cells (Mendrinna and Persson, 2015).

Coupling of cellulose synthase complexes and CMTs to achieve cellulose microfibril deposition at right angles to the original wall would require modification of the CMT array. Consistent with this proposition is the reported concurrent CMT reorganization with ingrowth wall construction during transfer-cell *trans*-differentiation of placental cells of *Lilium longifolium* (Singh *et al.*, 1999) and of abaxial epidermal cells of *V. faba* cotyledons (Bulbert *et al.*, 1998). In the latter case, as wall ingrowth papillae deposition commenced, CMTs became polarized to the site of ingrowth wall construction before being randomized to surround developing wall ingrowth papillae. However, the precise role of the modified CMT array in directing the pattern of localized wall ingrowth papillae deposition remains unclear.

The results of this study confirmed that the aligned CMT array is reorganized to a randomized array during *trans*-differentiation of epidermal cells. The changes not apparent from earlier studies are that circular depletion zones appeared in the randomized CMT array. The temporal and spatial pattern of these depletion zones equated with that of deposition of wall ingrowth papillae. However, the latter remained unchanged when CMTs were depolymerized or stabilized, negating a regulatory role for CMTs in directing deposition of wall ingrowth papillae. Interestingly, manipulating cellulose biosynthesis and cytosolic Ca^2+^ concentration during *trans*-differentiation to a transfer-cell morphology established that cytosolic Ca^2+^ plumes, known to define loci for wall ingrowth papillae formation (Zhang *et al.*, 2015), were responsible for localized depolymerization of CMTs to form the depletion zones within the randomized CMT arrays.

## Materials and methods

### Plant growth conditions

*V. faba* L. cv. Fiord plants were raised under controlled environmental conditions according to Zhou *et al.* (2010).

### Cotyledon culture

Excised cotyledons were cultured aseptically on either a solid or liquid form of MS medium adjusted to 300 mOsmol kg^–1^ using betaine. The medium pH was adjusted to 5.8 with 1M KOH or HCl prior to sterilization. For cultures examining changes across 24h of *trans*-differentiation, a liquid MS medium was used. For mid- and long-term culture periods (i.e. 3 and 6 d), solid MS medium with 0.8% aqueous agar, 100mM sucrose and 62.5mM l-asparagine was used to ensure a sufficient supply of sugar and amino acids to support ongoing cell activities. Sister cotyledon pairs were divided between MS medium with and without specified pharmacological agents. For all culture periods specified above, the excised cotyledons were first cultured at 4 °C for 4h to allow the pharmacological agent to be taken up by the epidermal cells. Thereafter, transfer-cell *trans*-differentiation was initiated by transferring the cotyledon cultures to 26 °C for the specified period examined. Each pharmacological agent was applied in MS medium at a concentration that did not impact cell viability as verified by staining tissue sections of cultured cotyledons with 0.1% (w/v) tetrazolium blue for 20min. For the two longer culture periods, cotyledons were transferred to fresh plates daily.

### Visualizing cortical microtubules and cell walls using confocal laser-scanning microscopy

*V. faba* cotyledons were fixed for 3h at room temperature in 4% (w/v) paraformaldehyde, 0.1% (w/v) glutaraldehyde, 2mM CaCl_2_ and 5mM dithiothreitol buffered in 50mM piperazine-*N*,*N′*-bis(2-ethanesulfonic acid) (PIPES), pH 7.0. After fixation, cotyledons were washed three times for 10min each in PBS and excess liquid was removed. Epidermal strips were peeled from the adaxial surface of each cotyledon (Dibley *et al.*, 2009) and used to immunolabel CMT arrays as follows. The epidermal peels were transferred to a 96-well plate and blocked for 1h in 250 μl PBS with 1% (w/v) bovine serum albumin (PBS/BSA) at room temperature. After blocking, peels were incubated overnight at 4 °C with a mouse monoclonal anti-α-tubulin antibody (Sigma-Aldrich, USA), diluted 1:100 in PBS/BSA, washed six times for 10min each in PBS with 0.2% (v/v) Tween 20 (PBS/T) and incubated for a further 3h at room temperature in secondary antibody, goat anti-rat IgG conjugated to Alexa Fluor 488 (Molecular Probes, Eugene, OR, USA) diluted 1:100 in PBS/T. Secondary antibody was removed by six 10min washes with PBS/T. Some epidermal peels were post-stained with filtered 0.5% (w/v) aqueous Congo red (Sigma, Australia) for 1min to visualize cell walls. Peels were mounted on glass slides in an anti-fading buffer containing 180 μl of Mowiol 4-88 with 20 μl of 0.1% (w/v) phenylenediamine.

An Olympus FV1000 confocal laser-scanning microscope (CLSM) with diode-pumped solid-state lasers, combined with an acousto-optic tunable filter laser combiner, was used to visualize the adaxial epidermal cells. A 473nm laser (15 mW, power set to 20%) with a 510–550nm emission filter set was used to visualize CMT fluorescence with the gain of the photomultiplier tube set to 600V. For Congo red fluorescence, a 559nm laser (15 mW, power set to 20%) was selected, with the emission filter and photomultiplier gain set to 600–660nm and 700V, respectively. Using cell wall fluorescence as a reference point, the focal plane for imaging was positioned 200–300nm inward of the inner surface of the uniform wall. A ×60 oil-immersion lens (numerical aperture 1.25) was used.

### Visualizing cytosolic calcium

Cultured cotyledons were loaded with a membrane-permeable Ca^2+^-sensitive dye, Oregon Green BAPTA-1 acetoxymethyl ester (OGB-1) at a concentration of 20 µM, for 3h at 4 °C. The loaded cotyledons were then incubated on liquid MS medium for 2h at 26 °C to allow cleavage of the Oregon Green ester by cytosolic esterases, hence trapping the membrane-impermeant Oregon Green dye in the cell cytosol (Zhang *et al.*, 2015). Transverse hand-cut sections were then prepared and stained with 0.1% (w/v) tetrazolium blue (prepared in PBS buffer containing 100mM sucrose) for 20min to identify viable cells for microscope observations. Thereafter, the tissue sections were counterstained with 0.1% (w/v) Calcofluor White for 30 s to outline cell walls of adaxial epidermal cells, before being mounted in 200 µl of PBS buffer containing 100mM sucrose and visualized using an Olympus FV1000 CLSM. A 405nm UV laser with a 440–490nm emission filter set was used to visualize Calcofluor white fluorescence, while a 473nm laser with a 510–550nm emission filter set was used to visualize OGB-1 fluorescence.

### Visualizing wall ingrowth papillae by electron microscopy

#### Scanning electron microscopy (SEM) 

Protoplasts of cotyledon adaxial epidermal peels (Dibley *et al.*, 2009) were removed by washing in 2% (v/v) NaOCl for 3h at room temperature with hourly changes followed by three 10min washes in dH_2_O to remove the bleach. Peels then were placed in small steel cages and dehydrated at 4 °C through a 10% step-graded ethanol/dH_2_O series, changed at 30min intervals from 10% up to 100% ethanol. After holding in 100% ethanol at least overnight, peels were critical-point dried with liquid CO_2_ in a critical-point drier (Balzers Union, Liechtenstein). The dried peels were orientated outer face down on sticky carbon tabs to reveal the cytoplasmic face of their outer periclinal cell walls. Samples were sputter coated with gold to a thickness of 20nm in a sputter-coating unit (SPI Suppliers, USA) and viewed at 15kV with a Philips XL30 scanning electron microscope. Cells were scored for the presence/absence of wall ingrowth papillae. Cell numbers induced to form wall ingrowth papillae were expressed as a percentage of total cells scored (130–150 cells for each replicate cotyledon). From these data, the mean percentage of cells containing wall ingrowth papillae, or the mean percentage of cells at specified stages of wall ingrowth papillae development, were computed as described by Zhou *et al.* (2010).

#### Transmission electron microscopy (TEM) 

Cotyledons were cut into 2×2×1mm blocks and fixed in 3% (v/v) glutaraldehyde and 4% (w/v) paraformaldehyde with 10mM sucrose in 50mM PIPES (pH 6.8) for 4h on ice, followed by post-fixation overnight at 4 °C in 1% (w/v) osmium tetroxide (ProSciTech, Qld, Australia) in 50mM PIPES buffer. Tissue was dehydrated in ethanol (10% steps), infiltrated, and embedded in LR White resin. Ultrathin (70nm thick) sections collected on Formvar-coated nickel 1nm slot grids were stained with saturated uranyl acetate and lead citrate and viewed with a JEOL 1200 EX II electron microscope.

#### Statistical analyses 

Treatment effects on cell percentages with wall ingrowth papillae and CMT distribution patterns were analysed by performing paired *t*-tests to determine the statistical significance of treatments. All image analysis was performed with ImageJ v.1.31 (http://rsb.info.nih.gov/ij). Microtubule angles were measured from confocal images relative to the assumed long axis of a cell using the ‘angle’ tool. Five hundred angles were measured for each replicate (five images per replicate, 100 measurements per image). For CMT arrays, images taken by Olympus FluoView FV1000 CLSM were converted and analysed in FV10-ASW 4.0 viewer software and all converted images were cropped in Adobe^®^ Photoshop CS2 to give an appropriate size.

## Results

### Microtubules are reorganized during wall ingrowth papillae formation

To investigate whether CMT organization changed during *trans*-differentiation of cotyledon epidermal cells to a transfer-cell morphology, *V. faba* cotyledons were cultured for 24h. The cytoplasmic face of the outer periclinal wall of epidermal cells in adaxial peels of the cultured cotyledons was viewed to assess wall ingrowth papillae formation, and peels were immunolabelled to visualize the spatial organization of their CMT arrays.

**Fig. 1.**
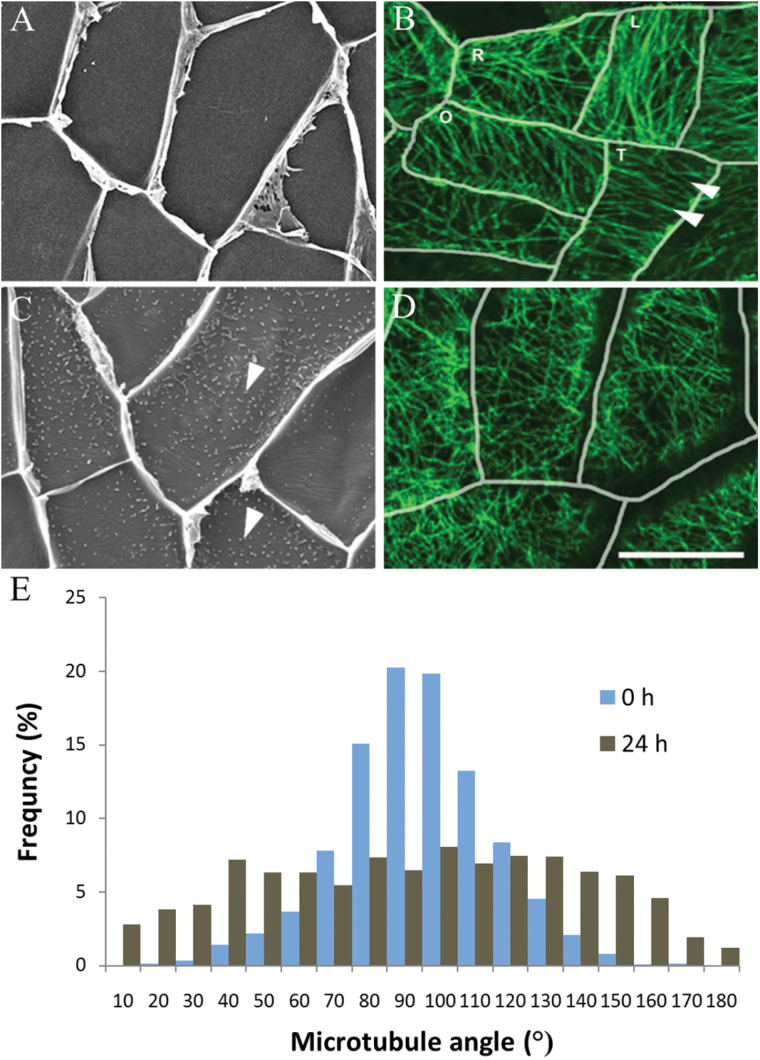
Changes in CMT organization during wall ingrowth papillae formation in adaxial epidermal cells of *V. faba* cotyledons cultured for 24h. (A–D) Adaxial epidermal peels from freshly harvested (0h) (A, B) or cotyledons cultured for 24h (C, D). Wall ingrowth papillae were visualized by viewing the cytoplasmic face of the outer periclinal wall of cells by SEM (A, C) or epidermal peels were immunolabelled with anti-α-tubulin and IgG–Alexa Fluor 488 conjugate to visualize CMT organization by CLSM (B, D). In freshly harvested cotyledons, CMT arrays (B, arrowheads) in their adaxial epidermal cells were mostly aligned in parallel arrays either transverse to the long (L) or short (T) axis of each epidermal cell, or in an oblique pattern to the long axis (O) (see E). In a small number of cells, the CMT array was organized randomly (R). After 24h of cotyledon culture, in which wall ingrowth papillae had been deposited in most cells (C, arrowheads), CMTs were randomly organized (D) (see E). Bar, 20 μm. (E) Angles of CMTs relative to the long axis of the cell expressed as the percentage frequency of total CMT angles measured.

In freshly harvested (*t*=0h) cotyledons, where wall ingrowth papillae formation had not commenced (Fig. 1A), CMTs in most adaxial epidermal cells were organized into parallel arrays aligned either parallel or transverse to the long axis of each cell or occasionally more obliquely (Fig. 1B). In cotyledons cultured for 24h, cells exhibited extensive numbers of developing wall ingrowth papillae (Fig. 1C) and CMTs in most cells were randomized (Fig. 1D). To quantify this change in CMT organization, the angles of CMTs relative to the long axis of each cell were measured. In epidermal cells of freshly harvested cotyledons (*t*=0h), the percentage frequency of CMT angle was distributed normally around a modal value perpendicular to the long axis of each cell (Fig. 1E). In contrast, after 24h of cotyledon culture, CMT angle was distributed more evenly across a 180° plane (Fig. 1E), indicating that randomization of CMTs occurred during wall ingrowth papillae formation.

### Three distinct CMT arrays are evident during wall ingrowth papillae formation

**Fig. 2.**
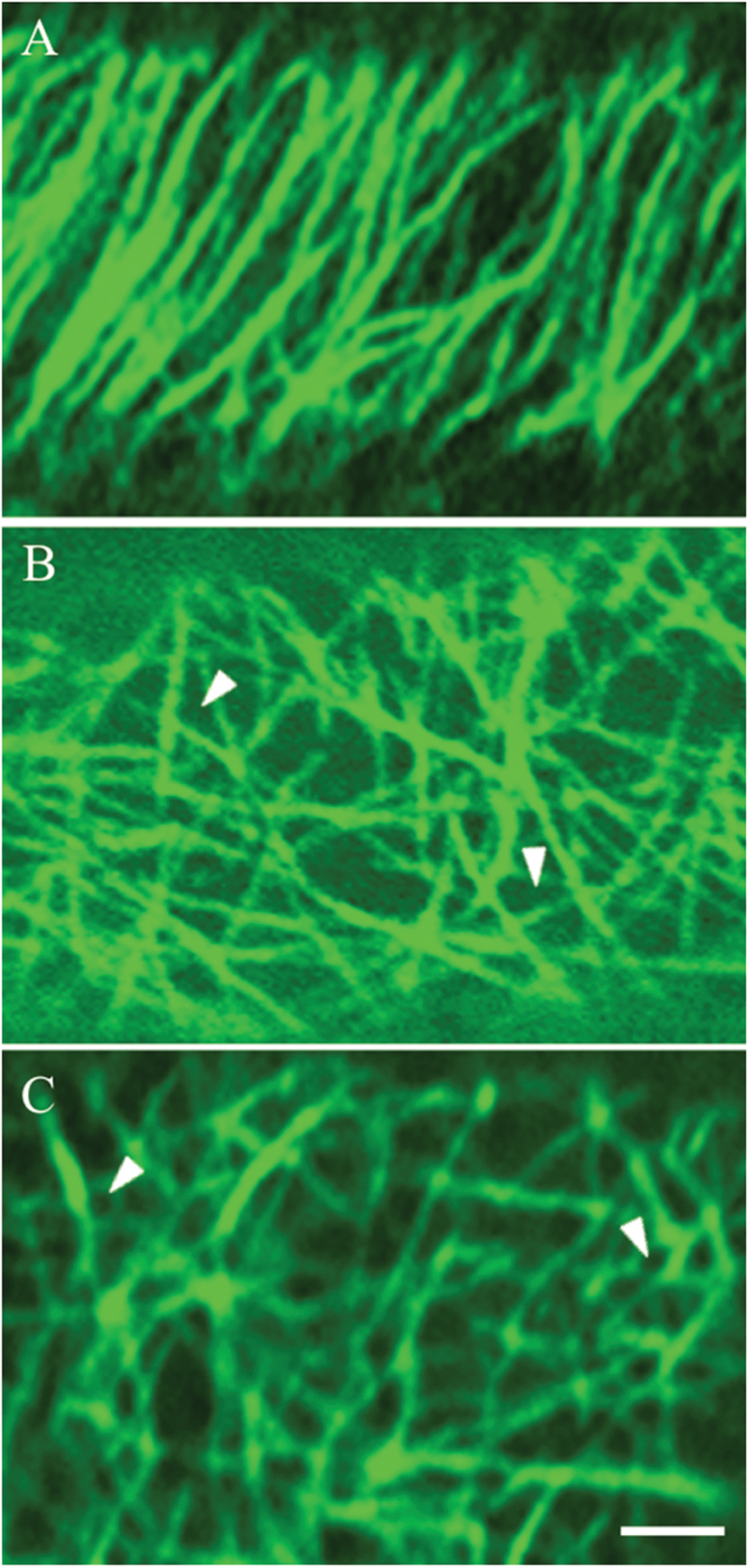
Confocal images of three categories of CMT arrays observed in adaxial epidermal cells of *V. faba* cotyledons cultured for 24h. CMT arrays immunolabelled with anti-α-tubulin and IgG–Alexa Fluor 488 conjugate. (A) ‘Organized’: parallel arrays of thick CMT bundles. (B) ‘Randomized’: an array defined by thick, strongly labelled CMT bundles arranged in a random network with distinctive polygonal gaps (arrowheads) in the network. (C) ‘Randomized with depletion zones’: an array composed of circular depletion zones surrounded by a possible combination of fine fragmented CMTs and tubulin monomers sometimes appearing like a collar (arrowheads). Bar, 2.5 μm.

Three different CMT arrays were identified to occur across 24h of cotyledon culture. These have been defined as ‘organized’, ‘randomized’, and ‘randomized with depletion zones’ (Fig. 2). ‘Organized’ arrays are composed of parallel thick CMT bundles characteristic of those found in expanding plant cells (Fig. 2A; see also Deinum and Mulder, 2013). In ‘randomized’ arrays, criss-crossing bundles of CMTs formed polygonal gaps in the CMT array (Fig. 2B). The ‘randomized with depletion zones’ arrays were composed of small circular depletion zones (terminology adopted from Oda *et al.*, 2010; Hardham, 2013). These depletion zones were surrounded by a possible combination of fine fragmented CMTs and tubulin monomers sometimes appearing like a collar (Fig. 2C). The striking difference between the last two CMT arrays (Fig. 2B, C) was that the fluorescence signal appeared to surround the circular depletion zones (Fig. 2C), whereas polygonal gaps were formed in the randomized array as a result of more linearly but randomly organized CMTs (Fig. 2B).

### Temporal appearance of the ‘randomized with depletion zones’ CMT array and dimensions of ‘depletion zones’ correlate with those of wall ingrowth papillae

**Fig. 3.**
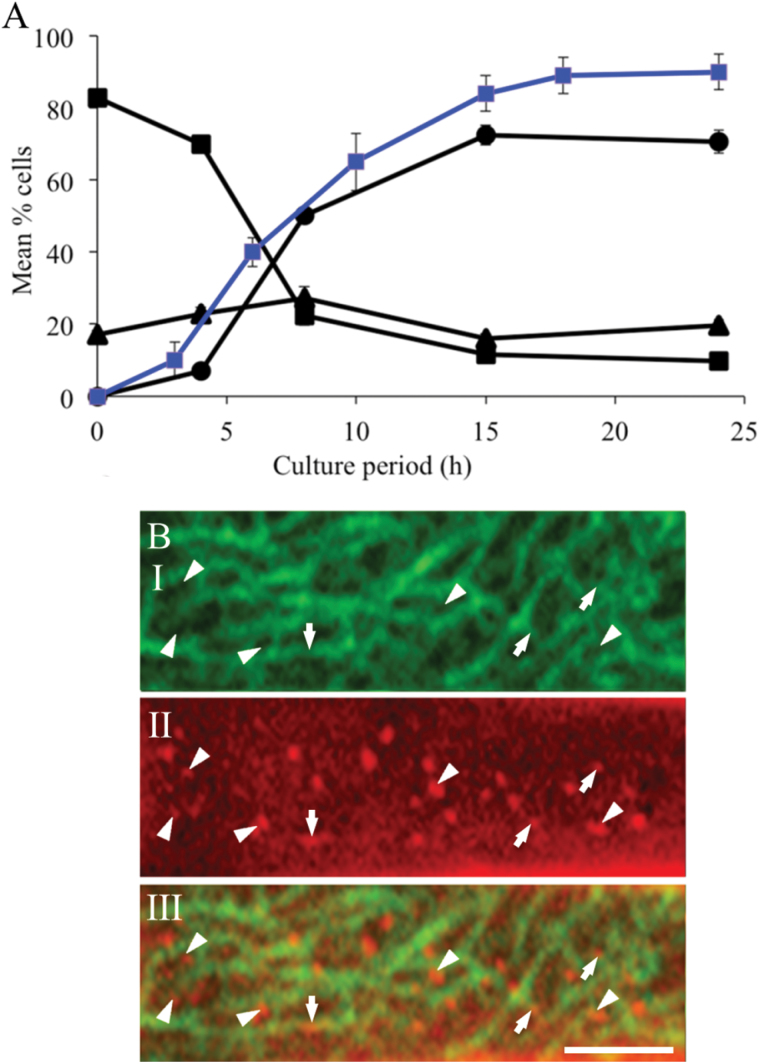
Temporal and spatial relationships between categories of CMT arrays and wall ingrowth papillae in adaxial epidermal cells of cultured *V. faba* cotyledons. (A) Temporal pattern of changes in the percentages of cells exhibiting organized (squares), randomized (triangles), and randomized with depletion zones (circles) CMT arrays across 24h of cotyledon culture. Data are means±SE from four replicate cotyledons with a minimum of 100 cells recorded for each cotyledon. Data from Wardini *et al.* (2007) on the percentage of epidermal cells with wall ingrowth papillae is superimposed (blue line). (B) Spatial relationship between circular depletion zones in the ‘randomized with depletion zones’ CMT array and wall ingrowth papillae in cells of cotyledons cultured for 24h. (I) CMTs immunolabelled with anti-α-tubulin and IgG–Alexa Fluor 488 conjugate. (II) Cell walls stained with Congo red. (III) Digital overlay of labelling of CMT array and cell wall staining. Circular depletion zones (I) intensely stained wall ingrowth papillae (II) and their co-localization (III) indicated by arrowheads. Arrows indicate shorter wall ingrowth papillae and their overarching CMTs. Bar, 2 μm.

To establish the temporal progression of the three CMT arrays (Fig. 2) in adaxial epidermal cells during wall ingrowth papillae formation, cotyledons were cultured for 0, 4, 8, 15, and 24h and the percentage of epidermal cells displaying each category of CMT array was determined (Fig. 3A). Prior to culture, over 80% of the epidermal cells displayed an ‘organized’ CMT array. By 4h of culture, cells with an ‘organized’ array were reduced to 70% as CMTs became ‘randomized’, and in a small percentage of cells, CMT arrays with ‘randomized with depletion zones’ were observed (Fig. 3A). By 8h of culture, a rapid decline in cells exhibiting ‘organized’ arrays to 20% was mirrored by an increase to 55% in cells displaying the ‘randomized with depletion zones’ CMT array (Fig. 3A). Thereafter, percentages of cells exhibiting the three categories of CMT arrays reached steady-state levels by 15h of cotyledon culture (Fig. 3A). Most significantly, the temporal appearance of the ‘randomized with depletion zones’ CMT array correlated strongly (*r* ^2^>0.95) with that of wall ingrowth papillae (Wardini *et al.*, 2007) across 24h of cotyledon culture (Fig. 3A).

The spatial relationship between the ‘randomized with depletion zones’ CMT array and wall ingrowth papillae was evaluated as follows. Epidermal peels of cotyledons cultured for 24h were immunolabelled to visualize CMT arrays and co-stained with Congo red. The latter stain preferentially binds cellulose (Meloan and Puchtler, 1978), permitting visualization of wall ingrowth papillae (Zhang *et al.*, 2015). Regions of intense Congo red staining (Fig. 3B, panel II) were found to co-localize with depletion zones in the CMT array (Fig. 3B, panel III), and in overlays, these represented 72±5% (*n*=10 overlays) of the total number of wall ingrowth papillae observed. The remaining 28% of wall ingrowth papillae appeared less strongly stained and were overarched by CMTs. This is consistent with these wall ingrowth papillae being shorter (<300 vs 500nm in length with the focal plane set between 200 and 300nm; see Materials and methods) and not yet extended through the CMT array (Fig. 3B, panel III). Additionally, the diameters of the depletion zones (488±9nm; *n*=800) were commensurate with those of the Congo red regions (500±19nm; *n*=800) and the basal regions of wall ingrowth papillae (430±8nm; *n*=240) measured from transmission electron micrographs. The 14% larger diameters of depletion zones and Congo red regions compared with diameters of wall ingrowth papillae probably resulted from a combination of tissue shrinkage during preparation for transmission electron microscopy and flaring of the fluorescent signal seen in confocal images. Collectively, these observations led to the hypothesis that wall ingrowth papillae formation is regulated by CMT network reorganization into a randomized array characterized by depletion zones within which the wall ingrowth papillae are located (Fig. 3B).

### Depolymerizing or stabilizing CMT arrays does not alter ingrowth wall papillae formation

The causality of the relationship between CMT reorganization and wall ingrowth papillae formation was tested by culturing cotyledons in the presence or absence of 20 µM oryzalin to depolymerize (Bajer and Molè-Bajer, 1986) or 5 µM taxol to stabilize (Schiff and Horwitz, 1980) the CMTs.

#### Effects over 24h of cotyledon culture

**Fig. 4.**
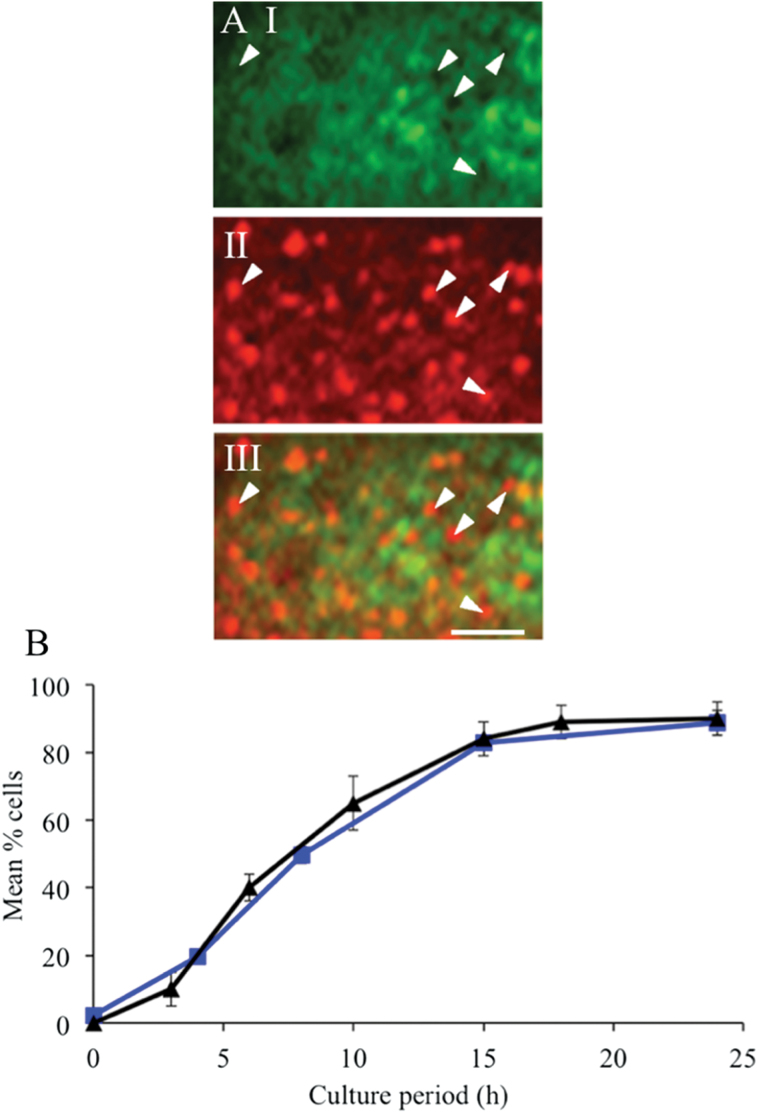
Effect of oryzalin on the spatiotemporal relationships of CMT arrays with wall ingrowth papillae in adaxial epidermal cells of cultured *V. faba* cotyledons. (A) Spatial relationship between circular depletion zones in the depolymerized aggregated α-tubulin fluorescence signal and wall ingrowth papillae in cells of cotyledons cultured for 24h. (I) Immunolabelled α-tubulin. (II) Congo red staining. (III) Overlay of the two channels. Circular depletion zones and wall ingrowth papillae are indicated by arrowheads. Bar, 2 μm. (B) Percentage of epidermal cells exhibiting circular ‘depletion zones’ within depolymerized tubulin (squares) and wall ingrowth papillae (triangles) (from Wardini *et al.*, 2007) across 24h of cotyledon culture. Results are means±SE derived from four replicate cotyledons with a minimum of 100 cells scored in each replicate.

After culturing cotyledons at 4 °C for 4h in the presence of oryzalin (*t*=0h), the CMT arrays were disrupted completely in over 80% of epidermal cells and ‘partially disrupted’ in the remaining cells (Supplementary Fig. S1, available at *JXB* online). After transferring oryzalin-treated cotyledons to 26 °C for 4h in the continued presence of oryzalin, CMTs were completely disrupted in 95% of cells (Supplementary Fig. S2E, F, available at *JXB* online) and remained so throughout the 24h culture period (Supplementary Fig. S2H, I, K, L, N, O). Thus, throughout the phase of wall ingrowth papillae deposition, the CMTs were disrupted. However, the anti-tubulin fluorescence appeared patchy, indicating that tubulin was aggregated (Figs 4A, panel I, and 5C, and Supplementary Fig. S2F, I, L, O).

**Fig. 5.**
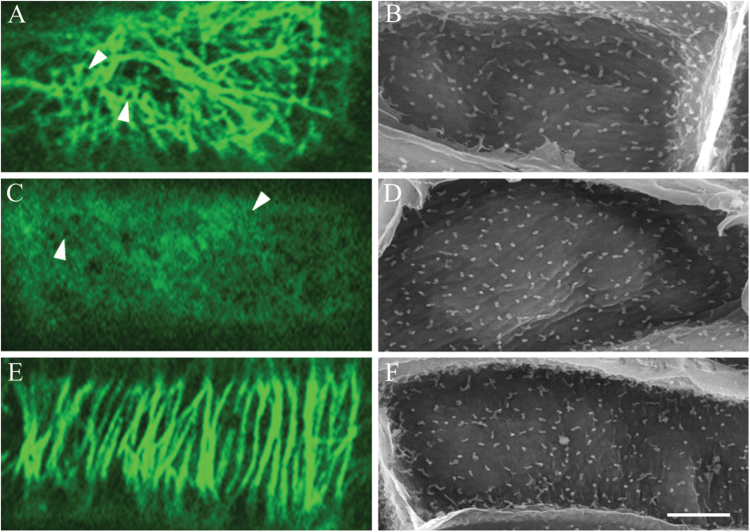
Effect of oryzalin and taxol on CMT array and wall ingrowth papillae formation in adaxial epidermal cells of cultured *V. faba* cotyledons. Confocal images of CMTs immunolabelled with anti-α-tubulin and IgG–Alexa Fluor 488 conjugate (A, C, E) and SEM images of the cytoplasmic face of the outer periclinal wall to visualize wall ingrowth papillae (B, D, F) of epidermal cells of cotyledons cultured for 24h in MS medium alone (A, B) or 24h in the presence of, 20 µM oryzalin (C, D) or 5 µM taxol (E, F). In the presence of oryzalin, CMT arrays were depolymerized and then tubulin aggregated and exhibited depletion zones (arrowheads in C). In contrast, CMT arrays were stabilized in the presence of taxol (E) retaining an alignment comparable to those located in freshly harvested cotyledons (c.f. Fig. 2A). Bar, 10 µm.

Of most interest to this study was the appearance of depletion zones within this patchy fluorescence (Figs 4A, panel I, and 5C and Supplementary Fig. S2I, L, O). These depletion zones were structurally similar, and had equivalent diameters, to those of the ‘randomized with depletion zones’ CMT array in epidermal cells of cotyledons cultured on MS medium alone (475 vs 488nm, respectively) (Fig. 2C; c.f. Fig. 4A, panel I). Co-staining immunolabelled epidermal peels of 24h cultured and oryzalin-treated cotyledons with Congo red established that these depletion zones co-localized with regions of intense Congo red staining (Fig. 4A, panel III). A similar pattern was detected for the depletion zones of cells of 24h cultured control cotyledons (Fig. 4A; c.f. Fig. 3B). Significantly, the temporal appearance of these depletion zones strongly correlated with that of wall ingrowth papillae (Fig. 4B; *r* ^2^=0.99) and of the depletion zones of control cotyledons (Fig. 3A; *r* ^2^>0.95). Moreover, the percentage of cells of oryzalin-treated cotyledons exhibiting wall ingrowth papillae was equivalent to that of the controls (Fig. 4B), as were wall ingrowth papillae densities (Table 1) and their morphology (Fig. 5D vs B). An interesting observation for which currently we have no explanation is that in 13.5±1.6% of cells of oryzalin-treated cotyledons, 1.5% of wall ingrowth papillae in these cells exhibited an aberrant organization. This small percentage of aberrant wall ingrowth papillae was apparent after 24h of culture and persisted in cotyledons cultured for 3 and 6 d (Supplementary Fig. S3, available at *JXB* online)

**Table 1.** Effect of CMT polymerization state, presence of cytosolic Ca^2+^ plumes, and cellulose biosynthesis on formation of depletion zones in CMT arrays and wall ingrowth papillae in adaxial epidermal cells of V. faba cotyledons Cotyledons were cultured for 24h in liquid MS medium in the absence/presence of oryzalin, taxol, BAPTA, DCB, and Eosin Yellow. Data are means±SE of 400 cells from four replicate cotyledons exposed to each treatment. For wall ingrowth papillae (WIP) density, data are means±SE of 100 cells from four replicate cotyledons.

Treatment	Cells with depletion zones (%)	Cells with WIPs	Density of WIPs per 100 μm^2^
Control	81.2±3.3	87.9±4.1	35.2±2.6
Oryzalin (20 μM)	82.7±3.4	86.3±6.8	39.4±4.2
Taxol (5 μM)	2.5±0.3	88.0±4.7	35.9±1.8
BAPTA (600 μM)	16.1±2.6	24.3±2.0	3.3±0.5
DCB (5 μM)	78.1±1.0	19.5±2.3	3.5±0.6
Eosin Yellow (0.5 μM)	1.3±0.4	17.6±1.4	1.7±0.7

CMTs in most *trans*-differentiating epidermal cells of cotyledons cultured on MS medium containing taxol were maintained as ‘organized’ arrays (Fig. 5E) with only 2.5% of cells exhibiting a ‘randomized with depletion zones’ array (Table 1). These data indicated that randomization of CMT arrays during the 24h culture period was slowed significantly, if not stalled, in taxol-treated cotyledons. However, control and taxol-treated cotyledons exhibited identical percentages of cells with wall ingrowth papillae (Table 1), as well as comparable wall ingrowth papillae densities (Table 1) and morphologies (Fig. 5F vs B). These results demonstrated that stabilization of CMT arrays did not affect the formation of wall ingrowth papillae.

#### Effects over 3 and 6 d of cotyledon culture

Wall ingrowth deposition in cotyledon epidermal cells involves sequential deposition of wall ingrowth papillae that branch and fuse to form a fenestrated layer of wall material. This sequence of deposition is repeated to form a multiple-layered ingrowth wall (Talbot *et al.*, 2001). To test if CMTs were required for the later stages of ingrowth wall formation, cotyledons were cultured for 4h at 4 °C in the absence/presence of either oryzalin or taxol, before culturing for 3 or 6 d at 26 °C in the absence/presence of the drug.

**Fig. 6.**
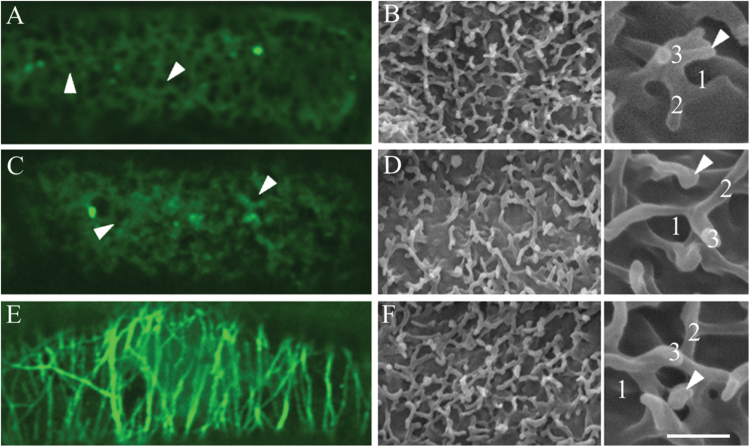
Effect of prolonged depolymerization or stabilization of CMT arrays on the formation of the ingrowth wall labyrinth in adaxial epidermal cells of *V. faba* cotyledons. Confocal images of immunolabelling with anti-α-tubulin and IgG–Alexa Fluor 488 conjugate (A, C, E) and SEM images of the cytoplasmic face of the ingrowth wall labyrinth (B, D, F) of epidermal cells of cotyledons cultured for 3 d on MS medium alone (A, B) or in the presence of 20 µM oryzalin (C, D) or 5 µM taxol (E, F). Note that in the control (A), the fluorescence is diffuse and there are some depletion zones (arrowheads). In oryzalin-treated cells (C), the tubulin fluorescence appeared to be aggregated in places, with depletion zones (arrowheads) visible, while in cells of the taxol-treated cotyledons (E), thick microtubule bundles were aligned perpendicular to their longitudinal axis. The ingrowth walls in (B), (D), and (F) were identical, being comprised of three fenestrated layers (number of layers labelled in inserts of B, D, F) with wall ingrowth papillae (arrowheads) arising from the most recently deposited fenestrated layer. Bar, 10 µm (A–F); 2 µm, inserts.

For cotyledons cultured on MS medium alone, the CMT array in over 95% of adaxial epidermal cells was randomized at 3 d (Table 2) and visualized as a diffuse fluorescence (Fig. 6A). Within this fluorescence, depletion zones equivalent to those of 24h cultured cotyledons (Fig. 5A) were evident, consistent with new papillae arising from the most recently formed fenestrated layer of the ingrowth wall (Fig. 6B). As the number of fenestrated layers increased from two to five with the longer (6 d) culture period (Table 2), the intensity of the fluorescence decreased (data not shown). In the presence of oryzalin, CMTs were completely depolymerized, and depletion zones, similar to those of control cells, were evident in the diffuse tubulin fluorescence by 3 d of culture (Fig. 6C) and remained unchanged over the longer period (data not shown). In contrast, in the majority of taxol-treated cells, thick bundles of CMTs were aligned perpendicular to the longitudinal axis of the epidermal cells (Fig. 6E) with this CMT organization being retained at 6 d (data not shown). For both oryzalin- and taxol-treated cotyledons, the percentages of cells with a fenestrated wall labyrinth (Table 2, Fig. 6D, F vs B), and the number of fenestrated layers forming the labyrinth (Table 2), were identical to that of the control at both 3 and 6 d of cotyledon culture. This finding indicated that polymerization or stabilization of CMT arrays had no discernible effect on fenestration of the ingrowth wall.

**Table 2.** Long-term effect of oryzalin or taxol on CMT organization and ingrowth wall formation in adaxial epidermal cells of V. faba cotyledons Cotyledons were cultured for 3 or 6 d on MS medium in the absence/presence of these pharmacological reagents. Data are means±SE of 400 cells from four replicate cotyledons exposed to each treatment. For layers of fenestrated wall ingrowth (WI), data are means±SE of 100 cells from four replicate cotyledons.

Culture time	Treatment	Cells with depletion zones (%)	Cells with fenestrated WI (%)	Number of WI layers
3 d	Control	97.2±2.4	83.8±5.4	2.8±0.1
	Oryzalin (20 μM)	98.5±1.1	81.4±2.1	2.8±0.2
	Taxol (5 μM)	15.4±1.2	81.1±2.2	2.7±0.1
6 d	Control	97.0±0.7	95.7±1.6	4.7±0.2
	Oryzalin (20 μM)	99.1±1.2	96.2±2.2	4.9±0.3
	Taxol (5 μM)	36.7±1.4	97.7±1.3	4.6±0.2

### Cytosolic Ca^2+^ regulates the spatiotemporal relationship between CMT depletion zones and deposition of wall ingrowth papillae

While the above results precluded a regulatory role for CMTs in wall ingrowth papillae deposition, CMT arrays were reorganized and depletion zones appeared in the CMT network during the first 24h of *trans*-differentiation of epidermal cells to a transfer-cell morphology (Figs 1 and 2). Furthermore, there was a correlation between the temporal appearance (Figs 3A and 4B), spatial localization (Figs. 3B and 4A), and the dimensions of these depletion zones and those of wall ingrowth papillae in both control and oryzalin-treated cotyledons. Given this close association, we explored the inter-relationship between appearance of the depletion zones within the CMT arrays, deposition of wall ingrowth papillae, and cytosolic Ca^2+^ plumes that define loci for wall ingrowth papillae deposition (Zhang *et al.*, 2015). Cotyledons were cultured for 24h in the presence of 600 µM 1,2-bis(*o*-aminophenoxy)ethane- *N*,*N*,*N’*,*N’*-tetraacetic acid (BAPTA), a Ca^2+^ chelator (Oiki *et al.*, 1994), 5 µM 2,6-dichlorobenzonitrile (DCB), a cellulose biosynthesis inhibitor (Montezinos and Delmer, 1980), or 0.5 µM Eosin Yellow, a Ca^2+^-ATPase inhibitor (De Michelis *et al.*, 1993).

**Fig. 7.**
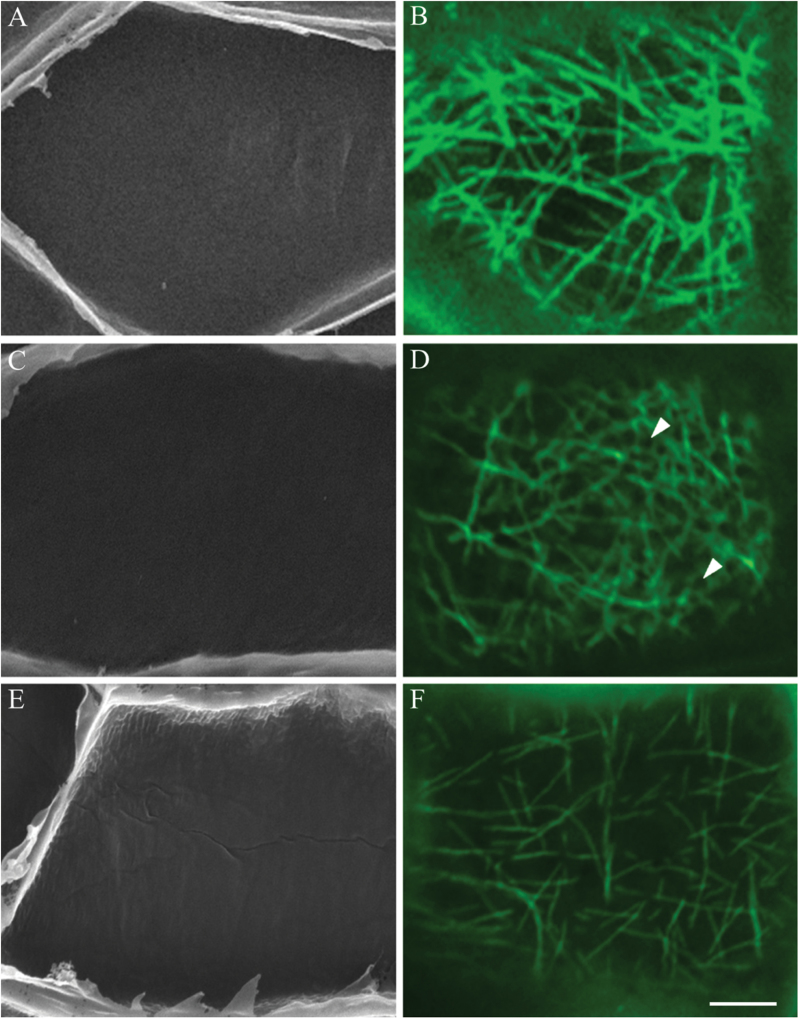
Effect of manipulating wall ingrowth papillae deposition and cytosolic Ca^2+^ on reorganization of CMT arrays in adaxial epidermal cells of cultured *V. faba* cotyledons. Representative SEM images of the cytoplasmic face of the outer periclinal wall (A, C, E) and confocal images of CMTs immunolabelled with anti-α-tubulin and IgG–Alexa Fluor 488 conjugate (B, D, F) of cells of cotyledons cultured for 24h in the presence of 600 µM of the Ca^2+^ chelator BAPTA (A, B), 5 µM of the cellulose biosynthesis inhibitor DCB (C, D) or 0.5 µM of the Ca^2+^ ATPase inhibitor Eosin Yellow (E, F). In the presence of BAPTA, wall ingrowth papillae formation was inhibited (A) and the CMT arrays, while randomized, were devoid of localized depletion zones (B). In contrast, when wall ingrowth papillae formation was inhibited in the presence of DCB (C), randomized CMT arrays exhibited circular depletion zones (D, arrowheads). Dissipation of localized Ca^2+^ plumes with Eosin Yellow resulted in a partially, but uniformly, depleted CMT array (F) and inhibition of wall ingrowth papillae formation (E). Bar, 5 µm.

In the presence of BAPTA, which inhibits the formation of cytosolic Ca^2+^ plumes and wall ingrowth papillae (Fig. 7A; Zhang *et al.*, 2015), no depletion zones occurred in the randomized CMT array (Fig. 7B) in 84 % of the epidermal cells (Table 1). This percentage equated to that of cells without wall ingrowth papillae (Table 1). In contrast, in the presence of DCB, depletion zones were evident in the randomized CMT array (Fig. 7D) in 78% of cells (Table 1). Assessing wall ingrowth papillae formation by SEM (Fig. 7C) established that deposition of wall ingrowth papillae had been substantially inhibited by DCB (Table 1; see also Talbot *et al.* 2007*a*). However, generation of the cytosolic Ca^2+^ signal (Zhang *et al.*, 2015) was unaffected by DCB (Supplementary Fig. S4B vs A, available at *JXB* online). These data suggested that the cytosolic Ca^2+^ plumes, and not deposition of wall ingrowth papillae, were responsible for forming the depletion zones within the CMT arrays. This conclusion was tested further under conditions where the cytosolic Ca^2+^ plumes were dissipated but elevated cytosolic concentrations were retained by culturing cotyledons in the presence of Eosin Yellow (Zhang *et al.*, 2015). While CMT arrays were partially depolymerized, no depletion zones were evident in these arrays (Fig. 7F) and the percentage of cells with wall ingrowth papillae was significantly decreased (Fig. 7E, Table 1). Collectively, these findings were consistent with the cytosolic Ca^2+^ plumes being responsible for the observed depletion zones that characterized the ‘randomized with depletion zones’ array.

## Discussion

### CMT reorganization during ingrowth wall formation results in a randomized CMT array with depletion zones

When *V. faba* cotyledons are placed in culture, their adaxial epidermal cells are induced spontaneously to undergo *trans*-differentiation to a transfer-cell morphology. A cohort of the epidermal cells immediately enters a phase of division (Dibley *et al.*, 2009). During this developmental phase, the parallel-aligned cortical arrays of microtubules present in the precursor epidermal cells were rapidly reorganized into a randomized CMT array (Figs 2B and 3A). In contrast to *trans*-differentiating epidermal cells, microtubule randomization in nematode-induced giant transfer cells was confined to those located in the cytoplasm, with CMTs remaining dense and filamentous (Kyndt *et al.*, 2013). Remodelling of cytoplasmic microtubules of giant cells is mediated by MAP65-3 (Caillaud *et al.*, 2008) and is associated with forming mitotic spindles and phragmoplasts, supporting a partial cytokinesis (Kyndt *et al.*, 2013).

As *trans*-differentiation of the epidermal cells progressed, circular depletion zones devoid of, but surrounded by, a possible combination of fine fragmented CMTs and tubulin monomers appeared within the randomized CMT arrays (Figs 1D and 2C), and by 6 d of culture, the tubulin fluorescence was diffuse (Fig. 6A). Depletion of CMT networks commonly occurs as cell wall deposition is completed (Sugimoto *et al.*, 2000). However, in the *trans*-differentiating epidermal cells, wall deposition continued for at least 6 d (Table 1), in the absence of recognizable microtubules (Fig. 6A). Of greater interest was a temporal increase in the proportion of *trans*-differentiating epidermal cells exhibiting circular depletion zones within their randomized CMT arrays (Fig. 3A). Microtubule-depletion zones have been observed in other physiological contexts. These include remodelled CMT arrays of cells developing into tracheary elements (Oda *et al.*, 2010) and as part of the defence response to invading pathogenic fungi or oomycetes. In the latter case, microtubule-depleted zones form in host cells immediately beneath the hyphal/appressorial contact sites (Hardham, 2013). Significantly, in both these systems the size of the depletion zone is consistent with the structure it defines. In differentiating tracheary elements, depletion zones and pits are ~5–10 µm in diameter (Oda *et al.*, 2010) while depletion zones of epidermal cells of *Arabidopsis* cotyledons resulting from mechanical stimulation that mimics fungal infection are 20 µm in diameter (Hardham *et al.*, 2008).

The temporal appearance of microtubule-depletion zones in CMT arrays of the *trans*-differentiating epidermal cells correlated with that exhibited by wall ingrowth papillae deposition (Fig. 3A). This finding, together with their comparable dimensions and most significantly their spatial inter-relationship (Fig. 3B), suggested that, during their construction, each wall ingrowth papilla extends into the cytoplasm through a microtubule-depletion zone. This relationship between microtubule-depletion zones and localized cell wall deposition differs from that reported for differentiating tracheary elements. In tracheary elements, the positioning of ‘pits’ in the secondary wall is due to exclusion of cellulose synthases by the microtubule depolymerizing enzyme MIDD1 (Oda *et al.*, 2010), which is guided to the cell wall by GTP-bound ROP11 (Oda and Fukuda, 2012, 2013*a*, *b*). Greater functional homology of the transfer-cell microtubule-depletion zones is shared with the defence response of plant cells to pathogenic fungi/oomycetes. Here, the pathogen-targeted cell deposits a localized wall papilla within the microtubule-depletion zone that impedes ingress of the pathogen into the host cytoplasm (Hardham, 2013).

### CMT array reorganization does not regulate wall ingrowth papillae deposition

Our results showed that, although there is a close spatiotemporal inter-relationship between cortical microtubules and wall ingrowth papillae, CMT arrays exerted no regulatory influence over either the pattern of papillae initiation (Table 1) or their morphology (Fig. 5D, F). This was demonstrated by depolymerizing or stabilizing microtubules by exposure of the *trans*-differentiating epidermal cells to oryzalin or taxol, respectively. Similarly, prolonged exposure to these drugs for up to 6 d did not affect deposition of the ingrowth wall labyrinth (Table 2, Fig. 6D, F). These responses differed from those reported for nematode giant cells. In giant cells, microtubule depolymerization with oryzalin reduced giant cell size, and stabilization with taxol slowed rates of giant cell initiation (de Almeida Engler *et al.*, 2004). These latter responses appeared to result from microtubule depolymerization/stabilization blocking nuclear division (de Almeida Engler *et al.*, 2004; Caillaud *et al.*, 2008). The absence of these responses in epidermal cells *trans*-differentiating to a transfer-cell morphology suggests that cell division exerts minimal influence over developmental events leading to deposition of wall ingrowth papillae. Similarly, construction of wall papillae to oppose invasion by pathogenic fungi/oomycetes was unaffected by a depolymerized or stabilized CMT array (Hardham, 2013).

Construction of flange wall ingrowths also exhibits a close relationship with microtubules whereby parallel arrays of microtubule bundles and cellulose microfibrils co-localize in xylem nodal transfer cells of *Triticum aestivum* and basal endosperm transfer cells of *Zea mays* (Talbot *et al.*, 2007*b*) in a pattern comparable to that found for microtubule-regulated secondary wall deposition (Baskin, 2001). Whether CMTs function in flange ingrowth wall deposition as for secondary wall thickenings remains to be determined.

### Cytosolic Ca^2+^ plumes are responsible for depletion zones within the CMT array

We were surprised to discover that circular depletion zones appeared within the aggregated tubulin resulting from depolymerized CMTs in cells cultured for 24h. The temporal appearance of these zones (Fig. 4B) and their dimensions equated with those of the depletion zones of control epidermal cells. Moreover, their spatial organization mirrored that of wall ingrowth papillae (Fig. 4A) in an identical configuration to that found for control epidermal cells (Fig. 4A; c.f. Fig. 3B). These findings raised the question as to the mechanism responsible for creating the CMT depletion zones. Broad possibilities included a mechanical force, generated by the inward-directed construction of wall ingrowth papillae into the cytosol, displacing the CMT array. Alternatively, the depleted-microtubule zones could be created by a mechanism that causes localized CMT depolymerization as described for developing tracheary elements (Oda and Fukuda, 2013a, *b*) or host cells invaded by pathogenic fungi/oomycetes (Hardham, 2013). For this scenario, CMT depolymerization would need to be closely co-ordinated in a spatiotemporal manner with construction of the wall ingrowth papillae. This could be achieved, for example, by a localized force on the plasma membrane imposed by each extending wall ingrowth papillae being transduced by a mechanosensitive mechanism to elicit a signal cascade leading to a localized depolymerization/remodelling of the CMT array (Hardham *et al.*, 2008).

To test these possibilities, construction of wall ingrowth papillae was inhibited by blocking cellulose biosynthesis with DCB (Table 1, Fig. 7C; see also Talbot *et al.*, 2007*a*). Under these conditions, microtubule-depletion zones still formed in the CMT network (Fig. 7D). This finding clearly dispels the concept that microtubule-depletion zones were formed as a result of an imposed mechanical force generated by the inward extension of developing wall ingrowth papillae displacing microtubules or on the plasma membrane to initiate a mechanosensitive signal cascade (Hardham, 2013). The possibility that wall ingrowth papillae deposition depended upon creation of localized zones of microtubule depolymerization, as found for formation of pits in differentiating tracheary elements (Oda *et al.*, 2010; Oda and Fukuda, 2012, 2013*a*, *b*), was also excluded as depolymerization of the CMT network by oryzalin (Fig. 5C) did not affect construction or patterning of wall ingrowth papillae (Table 1, Fig. 5D).

Plumes of cytosolic Ca^2+^ determine the loci at which wall ingrowth papillae are constructed in *trans*-differentiating epidermal cells (Zhang *et al.*, 2015). This raised the possibility that cytosolic Ca^2+^ plumes could provide a physiochemical environment to post-translationally regulate the catalytic activity of a microtubule-associated protein or proteins that function(s) to destabilize microtubules. In this context, we found that formation of the localized microtubule-depletion zones in epidermal cells was a Ca^2+^-dependent phenomenon, as evidenced by their appearance in DCB-treated cells being dependent on the presence of plumes of elevated cytosolic Ca^2+^ (Zhang *et al.*, 2015; see Fig. 7D vs B, Suppementary Fig. S3 and Table 1). Moreover, consistent with this conclusion was the observed loss of microtubule-depletion zones (Fig. 7F) under conditions where an elevated cytosolic Ca^2+^ concentration was sustained but the cytosolic Ca^2+^ plumes were dissipated by inhibiting Ca^2+^ efflux from the cytosol by an Eosin Yellow block of Ca^2+^-ATPase activity (Zhang *et al.*, 2015). Interestingly, this treatment resulted in a less dense CMT array composed of a randomly orientated meshwork of fine microtubules suggestive of partial microtubule depolymerization (Fig. 7F). The identity(ies) of the proposed Ca^2+^-dependent microtubule-destabilizing protein or proteins are not known. In other systems, microtubule-destabilizing protein25 (MDP25) has been shown to bind to and depolymerize microtubules following its Ca^2+^-dependent dissociation from the plasma membrane and release into the cytosol in senescing leaves (Keech *et al.*, 2010), elongating hypocotyls (Li *et al.*, 2011) and pollen tubes (Qin *et al.*, 2014). However, none of these examples describe formation of localized microtubule-depletion zones. This would rely on a mechanism to constrain the lateral spread of MDP25 through the cytosol as described for the formation of microtubule-depletion zones in differentiating tracheary elements (Oda and Fukuda, 2013*b*). To the best of our knowledge, localized depolymerization of CMTs by elevated cytosolic Ca^2+^ to form depletion zones within CMT arrays has not been reported previously.

### Possible role(s) of the microtubule-depletion zones in *trans*-differentiating transfer cells

Our findings raise the question of whether microtubule-depletion zones serve roles other than regulating patterns of cell wall deposition as found for differentiating tracheary elements (Oda *et al.*, 2010; Oda and Fukuda, 2012, 2013*a*, *b*). A hint that this may be the case is derived from the discovery that certain species-specific host/pathogen combinations, by blocking microtubule depolymerization/reorientation, lower resistance to pathogen invasion despite wall papillae forming beneath the pathogen contact site (Hardham, 2013).

One obvious possibility for transfer-cell development is that co-localization of microtubule-depletion zones with extending wall ingrowth papillae provides an unimpeded route for the papillae to grow through the CMT array into the cytosol. If this were not the case, the extending papillae, with centres of 1.4 µm and lengths of 500nm (Zhang *et al.*, 2015), could displace the CMT array 500nm into the cytoplasm away from contact with most of the plasma membrane lining the outer periclinal ingrowth wall. Avoiding this outcome by extension through depletion zones provides an uninterrupted contact for the CMT array with the plasma membrane lining the shanks of wall ingrowth papillae as well as cell wall regions between adjacent papillae. Organized in this way, the CMT array could play a key role in positioning transporters and thus facilitating sym-/apoplasmic exchange of nutrients and defence molecules across the plasma membrane abutting the ingrowth wall. Both of these phenomena represent core functions of transfer cells (Offler *et al.*, 2003).

As suggested for deposition of a wall papillae plug beneath pathogen contact sites (Hardham, 2013), we conclude that CMTs and cellulose synthase complexes are uncoupled (Paradez *et al.*, 2006) at the tips of developing wall ingrowth papillae. However, in contrast to other uncoupled systems such as tip growth (Mendrinna and Persson, 2015) the cellulose synthase complexes are constrained to the tips of developing wall ingrowths as they extrude whorls of cellulose microfibrils arranged perpendicular to the underlying uniform wall (Talbot *et al.*, 2007*a*). The mechanism constraining these cellulose synthase complexes is currently unknown but is likely to be a Ca^2+^-dependent process as shown by the plumes of elevated cytosolic calcium being essential for wall ingrowth formation (Zhang *et al.*, 2015).

## Supplementary data

Supplementary data are available at *JXB* online

Supplementary Fig. S1. Effect of the MT-disrupting drug oryzalin on CMT arrays in adaxial epidermal cells of cultured *V. faba* cotyledons.

Supplementary Fig. S2. Effect of the MT-disrupting drug oryzalin on CMT organization in adaxial epidermal cells of cultured *V. faba* cotyledons.

Supplementary Fig. S3. Aberrant wall ingrowth papillae in oryzalin-treated epidermal cells of cultured *V. faba* cotyledons

Supplementary Fig. S4. Effect of the cellulose biosynthesis inhibitor DCB on the generation of a polarized Ca^2+^ signal in adaxial epidermal cells of cultured *V. faba* cotyledons.

Supplementary Data

## Abbreviations:

BAPTA: 1,2-bis(*o*-aminophenoxy)ethane- *N*,*N*,*N’*,*N’*-tetraacetic acid
BSA: bovine serum albumin
CLSM: confocal laser-scanning microscope
CMT: cortical microtubule
DCB: 2,6-dichlorobenzonitrile
MS: Murishige and Skoog
OGB-1: Oregon Green BAPTA-1 acetoxymethyl ester
PBS: phosphate buffered saline
PIPES: piperazine-*N,N′*-bis (2-ethanesulfonic acid)
SE: standard error
SEM: scanning electron microscope
TEM: transmission electron microscopy.
