# Supplementary material for: Calcium-dependent depletion zones in the cortical microtubule array coincide with sites of, but do not regulate, wall ingrowth papillae deposition in epidermal transfer cells
Source: J Exp Bot. 2015 Jul 1;66(19):6021–33. doi: 10.1093/jxb/erv317 (PMC4566988; doi:10.1093/jxb/erv317)
Supplement: Supplementary Data [file supp_66_19_6021__index.html]

Calcium-dependent depletion zones in the cortical microtubule array coincide with sites of, but do not regulate, wall ingrowth papillae deposition in epidermal transfer cells — Calcium-dependent depletion zones in the cortical microtubule array coincide with sites of, but do not regulate, wall ingrowth papillae deposition in epidermal transfer cells — Supplementary Data 

# Calcium-dependent depletion zones in the cortical microtubule array coincide with sites of, but do not regulate, wall ingrowth papillae deposition in epidermal transfer cells

## Supplementary Data

Data files

- Supplementary Data - Supplementary Data
