# Supplementary material for: Calcium-dependent depletion zones in the cortical microtubule array coincide with sites of, but do not regulate, wall ingrowth papillae deposition in epidermal transfer cells
Source: J Exp Bot. 2015 Jul 1;66(19):6021–33. doi: 10.1093/jxb/erv317 (PMC4566988; doi:10.1093/jxb/erv317)
Supplement: Supplementary Data [file supp_erv317_jexbot148106_file001.pdf]

Supplementary material

Calcium-dependent depletion zones in the cortical microtubule array coincide with sites of, but do not regulate, wall ingrowth papillae deposition in epidermal transfer cells

Hui-ming Zhang, Mark J. Talbot, David W. McCurdy, John W. Patrick and Christina E. Offler

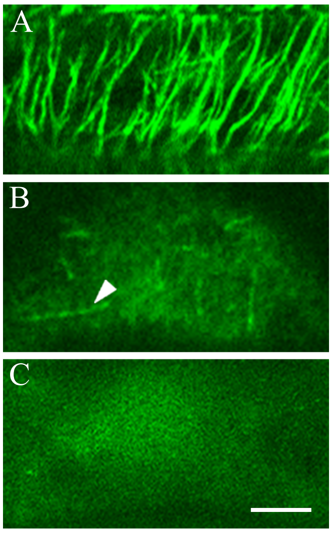

Figure S1. Effect of oryzalin on CMT arrays in adaxial epidermal cells of cultured *V. faba* cotyledons. Cotyledons were cultured in liquid MS medium for 4 h at 4 ºC, in the absence (A) or presence (B, C) of 20 μM oryzalin, before being transferred to 26 ºC and cultured for a further 0 (A, B) or 24 (C) h. Confocal images of CMTs immunolabelled with anti-α-tubulin and IgG Alexa488 conjugate illustrating three stages of CMT array deploymerization. (A) 'Intact'– CMTs are intact, strong bundles extending across most of the cell axis. (B) 'Partially depolymerized' - a few short CMT bundles remain (dart) amid a diffuse anti-α-tubulin fluorescence signal. (C) 'Depolymerized' - no CMT bundles evident and only a diffuse anti- α-tubulin fluorescence signal. Bar = 2.5 μm.

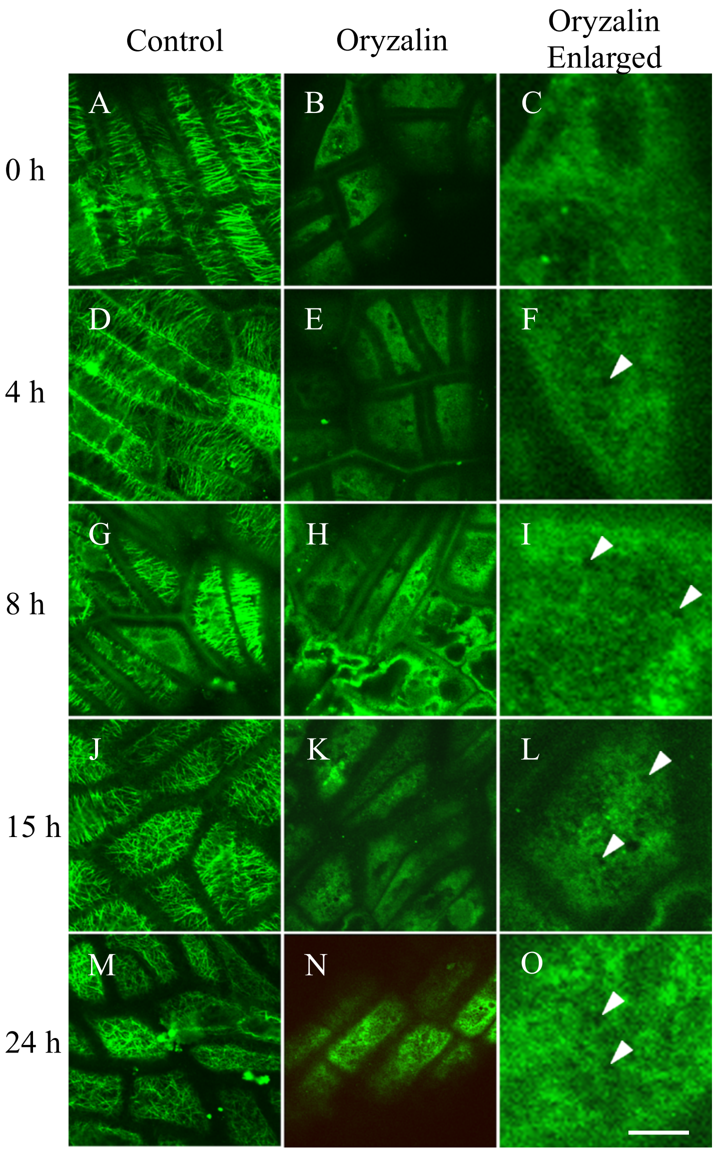

Figure S2. Effect of microtubule-disrupting drug oryzalin on CMT organization in adaxial epidermal cells of cultured *V. faba* cotyledons. Confocal images of anti-tubulin fluorescence (A - O). Cotyledons were cultured in liquid MS medium for 4 h at 4 ºC, in the absence (A, D, G, J, M) or presence (B, C, E, F, H, I, K, L N, O) of 20 µM oryzalin, before being transferred to 26 ºC and cultured for a further 0 (A - C), 4 (D – F), 8 (G - I), 15 (J - L) and 24 (M - O) h in the absence/presence of drug treatment. In the absence of oryzalin, the organization of CMT arrays in the epidermal cells gradually changed from an 'organized' array (A, D) to a 'randomized-depleted with collars' array (J, M). In the presence of oryzalin, by 4 h at 4 ºC the CMT arrays in most cells were completely disrupted and only a smeared tubulin fluorescence was detected (B, C). After cells were transferred to 26 ºC and cultured for a further 4 h in the presence of oryzalin, in about 10% of the cells, some localized deformation of

tubulin fluorescence resulting in non-fluorescent circles in the depolymerized smear of tubulin fluorescence were visualized (arrowhead in F). After exposure to oryzalin for 4 – 24 h, the proportion of cells with the 'non-fluorescent circles in depolymerized smear of tubulin fluorescence' increased (I, L, O) and the density of circles in the cells also increased (arrowheads in I, L, O). From 8 h on, the tubulin signal became patchy suggesting some degree of tubulin aggregation in epidermal cells in which WI papillae were being deposited. Bar = 20 μm.

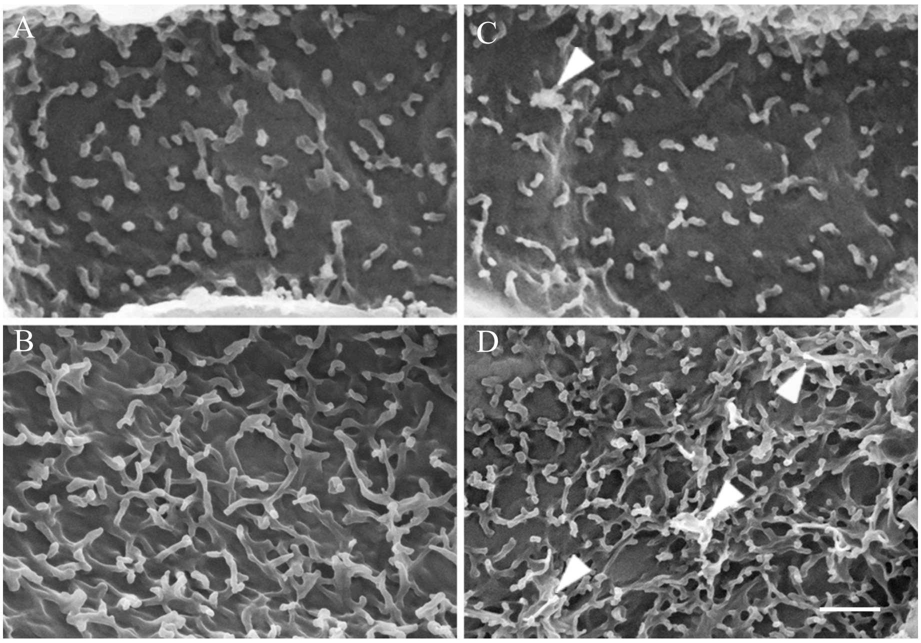

Figure S3. Aberrant wall ingrowth papillae structures formed in the presence of oryzalin in adaxial epidermal cells of *V. faba* cotyledons**.** Representative SEM images of the cytoplasmic face of the outer periclinal wall of adaxial epidermal cells of . cotyledons cultured in MS medium for 4 h at 4 ºC in the absence (A, C) or presence (B, D) of 20 µM oryzalin, then transferred to 26 ºC for a further 24 h (A, B) or 72 h (C, D) before being prepared for SEM. Note in cells with aberrant wall ingrowth papillae structures (darts in B, D), the remainder of the wall ingrowth papillae (B) and fenestrated layers (D) were equivalent in density and morphology to those of their matching controls (A cf B; C cf D). Bar = 2.5 µm.

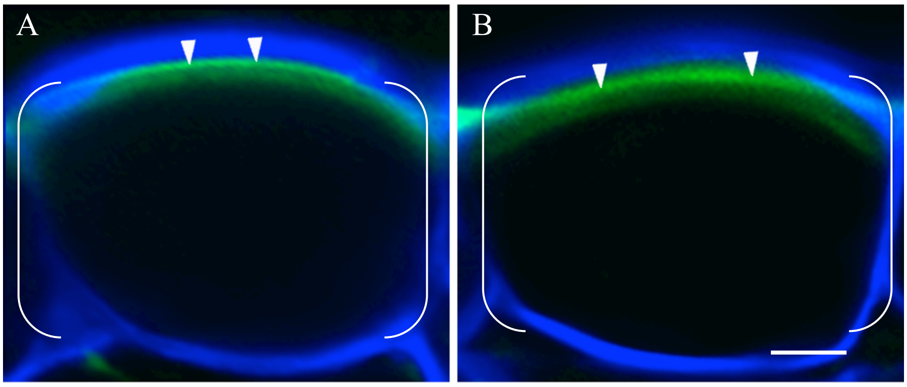

Figure S4. Effect of the cellulose biosynthesis inhibitor DCB on the generation of a polarized calcium signal in adaxial epidermal cells of cultured *V. faba* cotyledons. Confocal images of transverse hand-sections of adaxial epidermal cells (bracketed) cultured in the absence (A) and presence (B) of 5 μM DCB for 24 h prior to loading with 20 μM Ca^{2+}-sensitive dye, Oregon Green BAPTA-1 acetoxymethyl (AM) ester and counterstaining with 0.1% (w/v) Calcofluor White, the latter to identify cell walls (blue fluorescence). Note the Oregon Green fluorescence is localized inward of the outer periclinal wall of cells (darts) and is identical in both control (A) and DCB-treated (B) cotyledons. Bar = 2.5 μm.
